# Supplementary material for: Characterization of Cationic Modified Short Linear Glucan and Fabrication of Complex Nanoparticles with Low and High Methoxy Pectin
Source: Foods. 2021 Oct 19;10(10):2509. doi: 10.3390/foods10102509 (PMC8535971; doi:10.3390/foods10102509)
Supplement: Supplementary file 1 [file foods-10-02509-s001.zip › foods-1336043-supplementary.pdf]

Table S1. The enthalpy of the individual biopolymer and their nanocomplex particles.

Sample	ΔH (J/g)
LMP	138.56
LMP-CSLG 0.5	102.54
LMP-CSLG 1.0	113.62
LMP-CSLG 2.5	118.25
LMP-CSLG 5.0	129.63
HMP	145.34
HMP-CSLG 0.5	124.52
HMP-CSLG 1.0	129.58
HMP-CSLG 2.5	133.47
HMP-CSLG 5.0	138.62
CMCS	97.68
CMCS-CSLG 0.5	76.59
CMCS-CSLG 1.0	80.38
CMCS-CSLG 2.5	87.54
CMCS-CSLG 5.0	92.46
